# The protective effect and mechanism of rapamycin in the rat model of IgA nephropathy

**DOI:** 10.1080/0886022X.2019.1577257

**Published:** 2019-05-06

**Authors:** Ning Guo, Shengli Liu, Laurine M Bow, Xianquan Cui, Luwei Zhang, Shihao Xu, Sai Lu, Jun Tian

**Affiliations:** a Department of Organ Transplantation, Qilu Hospital, Shandong University, Jinan, China;; b Transplant Immunology Laboratory, Hartford Hospital, Hartford, CT, USA;; c Department of Transplantation Surgery, Yale University School of Medicine, New Haven, CT, USA

**Keywords:** IgA nephropathy, rapamycin, animal model, mTOR pathway

## Abstract

**Background:** The pathogenesis of the development of IgA nephropathy has not been clear up to now. At present, some studies revealed that the mTOR pathway may participate in IgA nephropathy; however, the mechanism has not been systematically studied. In this study, we established an IgAN rat model to investigate the protective effects of rapamycin as a new type of immunosuppressant, as well as its therapeutic mechanisms.

**Methods:** After the establishment of IgA nephropathy model, rats were treated with different concentrations of rapamycin, and the protective effect of different concentrations of rapamycin on renal function of the rats was observed. The deposition of IgA was observed by immunofluorescence. The kidney expression of Akt and p70S6k proteins in mTOR pathway was examined using the western blot assay after rapamycin treatment.

**Results:** Morphology and immunofluorescence confirmed that the rat model of IgA nephropathy was successfully established. In particular, the level of proteinuria decreased with the increase of the dose of rapamycin, as well as the deposition of IgA in glomeruli. Moreover, the western blot analysis indicated that the expression of p70S6K in the downstream of mTOR pathway decreased and the upstream protein AKT of the mTOR pathway was overexpressed in the rats model.

**Conclusion:** We found that rapamycin has protective effects in the IgA nephropathy rat model in a dose-dependent manner. In addition, the result of western blot assay suggested that rapamycin may display its therapeutic effects through interfering the AKT-mTOR-p70S6K signaling pathway.

## Introduction

At present, the pathophysiology of IgA nephropathy(IgAN) still remains unclear and may be related to many factors, such as infections, immune responses, inflammatory mediators, genetics, etc. [1,2]. Many studies suggested that the abnormal glycosylation of IgA molecules and the formation of immune complexes played important roles in the pathogenesis of IgAN [3]. Structurally, abnormal IgA can evade the phagocytosis of hepatocytes and monocytes in IgAN patients and deposit in the mesangium of glomeruli. In addition, IgA can bind to its receptors on mesangial cells and neutrophils, stimulating cell proliferation, and the release of a variety of cytokines which can promote cell re-proliferation and aggregation, leading to interstitial fibrosis eventually [4]. However, the treatment to interfere with the process of IgAN and its mechanism has not been intensively studied.

Rapamycin is a novel immune complex that can display immunosuppressive effects by inhibiting T-cell proliferation [5]. It has been proved that rapamycin can improve renal function, decrease the levels of proteinuria, alleviate renal tubulointerstitial infiltration, and interstitial fibrosis in IgAN [6]. In the mesangial proliferative rat model, rapamycin could significantly reduce the various symptoms of glomerulonephritis [7]. Meanwhile, low dose of rapamycin inhibited the proliferation of mesangial cells [8]. Therefore, we speculate that rapamycin may play an important role in the prevention and treatment of IgA nephropathy through its anti-inflammatory and anti-proliferative effects.

The target protein of rapamycin is mTOR, whose activity is influenced by a variety of regulatory signal proteins. The PI3K/Akt/mTOR signaling pathway can accelerate the cell life cycle, reduce apoptosis, and promote cell migration. In addition, various diseases may be related to the PI3K/Akt/mTOR signaling pathway [9]. Currently, studies showed that the mTOR pathway is overexpressed in children with IgAN, but the pathological mechanism had not been systematically studied [10]. Therefore, we established a rat model of IgAN to observe the therapeutical effects of rapamycin in the progression of IgAN and find possible mechanisms.

## Materials and methods

### Materials and reagents

The healthy and clean grade 6-week old SD male rats were bought from the animal experiment center of Shandong University, rabbit IgA polyclonal antibody was bought from biosynthesis company (Cat no:bs-10491r),4',6-diamidino-2-phenylindole (DAPI) was bought from Sigma-Aldrich company (Cat no: D954). Rapamycin was kindly provided by Hangzhou East China Pharmaceutical Co; rabbit AKT polyclonal antibody was bought from CST company (Cat no:9272S); rabbit p70S6K polyclonal antibody was bought from Fitzgerald company (Cat no:70R-36369); rabbit GAPDH monoclonal antibody was bought from CST company(Cat no:2118S).

### Methods

Sixty-six male Sprague − Dawley Rats of clean grade weighing 150 ± 10 g were purchased from Animal Experiment Center of Shandong University and were fed adaptively for 1 week. All of the rats showed active, shiny hair, and negative during the twice urine protein qualitative test. Animals were given free access to water and a standard laboratory diet in the whole experiment.

#### Preparation of IgAN model

Sprague − Dawley rats were intragastrically administered with bovine serum albumin (BSA, 400 mg/kg) every other day for 12 weeks. From the fourth week, 0.3 mL of castor oil and 0.1 mL of carbon tetrachloride was subcutaneously injected once a week for 12 weeks. The eighth week of lipopolysaccharide (LPS) 0.05 mg tail vein injection was injected once a week until the 12th week. The control group was given the same amount of steaming water every other day for 12 weeks, subcutaneous injection of 0.4 mL of sodium chloride solution once a week for 12 weeks, the same amount of sodium chloride solution was injected intravenously at the end of the eighth week. At the end of the 12th week, three rats in the control group and model group were taken respectively, whose frozen sections of renal cortex tissue were stained with periodic acid-Schiff (PAS). Direct immunofluorescence (FITC-conjugated rabbit anti-rat IgA antibody) was utilized to visualize IgA deposition in the glomerular mesangium by immunofluorescence microscopy.

The weights of rats in each group were measured every weekend, 24-h urine of the rats was collected by metabolism cage and 24-h urine proteinuria was observed on the weekends of 4, 8, 12, and 16. At the end of 16th week, the rats were anesthetized by 10% chloral hydrate and 3 mL of blood was collected from the abdominal cavity. After standing at room temperature for 30 min, the serum was separated from the blood samples by centrifugation as quickly as possible, automatic biochemical analyzer was utilized to detect the biochemical indicators. A portion of the kidney tissue was collected and fixed in 10% buffered formalin for light microscope observation and immunohistochemical detection. Some of the renal cortex were wrapped with tin box paper and quickly stored in liquid nitrogen for extraction of total protein in the kidney tissue subsequently.

#### Rapamycin intervention

At the 12th week, the rat were divided into control group, IgAN group, and the rapamycin treatment groups A–C by intragastric administration once every day (doses of rapamycin were 0.5 mg/kg/d(A), 1 mg/kg/d(B), 1.5 mg/kg/d(C)), each group contained 12 rats. The immunofluorescence assay was conducted to observe the changes of IgA deposition in the glomerular mesangium and the mTOR pathway related proteins were also detected to analyze the changes among different groups.

#### Immunofluorescence assay

The rats were divided into five groups as mentioned above. The slices were removed from the −70 °C refrigerator and reheated (placed at room temperature until there is no water vapor on the slice). The nail polish was painted around the slices and dried. The slices were washed with 0.1 M PBS for twice (5 min each time), and then washed with 1% TritonX-100 and 0.1 M PBS for 30 min, sealing for 1–2 h at room temperature. After being diluted and mixed up, immunohistochemistry primary antibody was added to the slices (on wet box), fixing at 4 °C for 24 h. The wet box was removed from the refrigerator, and the slices were washed with 0.1 M PBS for six times (10 min each time). The absorbent paper was utilized to soak up the nail polish and the water around it; fluorescent secondary antibody was added to the slices subsequently. After incubation for 2 h at room temperature and avoided light, we removed the wet box from the incubator and put the slices into the PBS box to wash for six times (5 min each time). The slices were washed with DAPI (1:1000) at the third time to dye the cell nucleus.

#### Western blot assay

Total protein was extracted according to the introduction of protein extraction kit. The concentrations of protein were determined and adjusted to equal concentration. The buffer was added to the protein extract and fixed up; the mixture was allowed to boil for 5 min. The target proteins were transferred to the membrane using the wet method and were sealed for 1 h at room temperature. Antibody-1 diluent was added to the membrane and incubated at 4 °C overnight, then adding Antibody-2 diluent and incubated at room temperature for 2 h. ECL chemiluminescence was used for visualization.

#### Statistical approach

Statistical analysis used SPSS 17.0 statistical software. Continuous variables are expressed as mean ± SE. Comparisons among groups were evaluated using variance analysis or Wilcoxon rank sum test. Differences were considered significant for *p* < 0.05.

## Results

**Figure 1.**
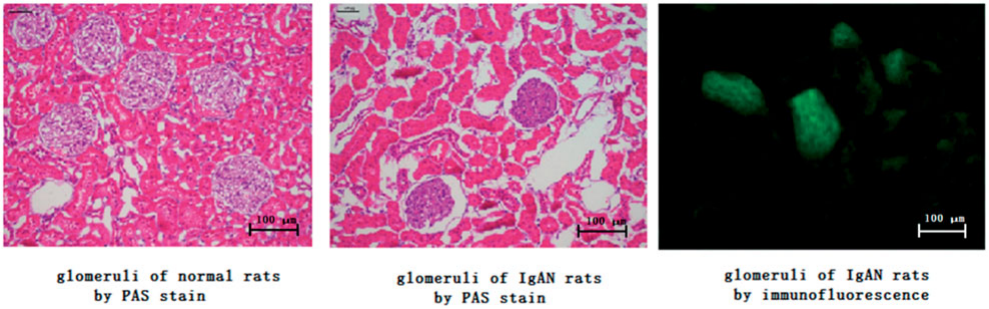
PAS stain and immunofluorescence photograph of IgAN rats model.

Under light microscope, the tissue structure of the rats in the control group was normal; the rats in the IgAN model groups displayed pathological changes at the 12th weekend. The glomeruli showed swelling and volume increase, especially in the mesangial cells and matrix, similar to the pathomorphological changes under light microscope in clinical practice. At the 12th weekend, continuous flake deposition of IgA was observed in the glomerular mesangium of rats in the IgAN model groups (Figure 1).

At the 12th weekend, comparison of the biochemical index except kidney functions (including TP, ALB, ALT, and AST) between the control group and model group showed no statistically significant difference (*p* > 0.05, Table 1). However, the model group had higher biochemical index of kidney functions(including Scr and Bun) than the control group(*p* < 0.05, Table 1), There was no statistical difference of the average body weight of rats between model group and control group during the whole experiment (*p* > 0.05, Table 2). The degree of proteinuria in IgAN model group was significantly higher than that in control group(*p* < 0.05 at fourth week point, *p* < 0.01 at eighth,12th, and 16th weed point, Table 3), and the degree of proteinuria gradually decreased along with the concentration of rapamycin treatment (Table 4).

**Table 1.** Function of kidney and liver of control group and IgAN group.

16 Weeks	Control	IgAN
Serum creatinine (umol/L)	31.23 ± 7.22	45.11 ± 6.02*
Urea nitrogen (mmol/L)	5.42 ± 1.31	8.05 ± 1.69*
Total protein (g/L)	56.38 ± 7.61	53. 75 ± 6.04
Albumin (g/L)	14.15 ± 1.61	12.55 ± 2.27
Alanine amino transferase(U/L)	37.75 ± 7.01	32.15 ± 5.51
Aspartate amino transferase(U/L)	82.13 ± 16.61	88.65 ± 17.58

*Showed there was statistical significance between 2 groups.

**Table 2.** The Rats weight of control group and IgAN group.

Unit: g	Pre	4 Weeks	8 Weeks	12 Weeks	16 Weeks
Control	148.1 ± 15.6	268.3 ± 37.2	338.7 ± 36.3	367.2 ± 33.2	451.3 ± 41.2
IgAN	156.3 ± 17.2	231.2 ± 17.2	312.2 ± 31.2	352.3 ± 3 7.6	442.2 ± 47.8

**Table 3.** The situation of proteinuria of control group and IgAN group.

Unit：mg/24h	Pre	4 weeks*	8 weeks**	12 weeks**	16 weeks**
Control	1.13 ± 0.17	2.31 ± 0.48	3.38 ± 0.36	4.13 ± 0.72	4.37 ± 0.41
IgAN	1.56 ± 0.12	6.16 ± 1.72	10.49 ± 2.21	13.78 ± 3.76	16.23 ± 4.28

*Showed there was statistical significance between 2 groups and *p* < 0.05.

**Showed there was statistical significance between 2 groups and *p* < 0.01.

**Table 4.** The situation of 24 h urine protein after treatment and the degree of proteinuria gradually decreased along with the concentration of rapamycin treatment.

24 h urine protein after treatment	Unit：mg/24 h
IgAN group	16.23 ± 4.28
0.5 mg/kg/d group	12.42 ± 2.31
1.0 mg/kg/d group	10.38 ± 1.93
1.5 mg/kg/d group	7.65 ± 1.61
Control group	4.37 ± 0.41

**Figure 2.**
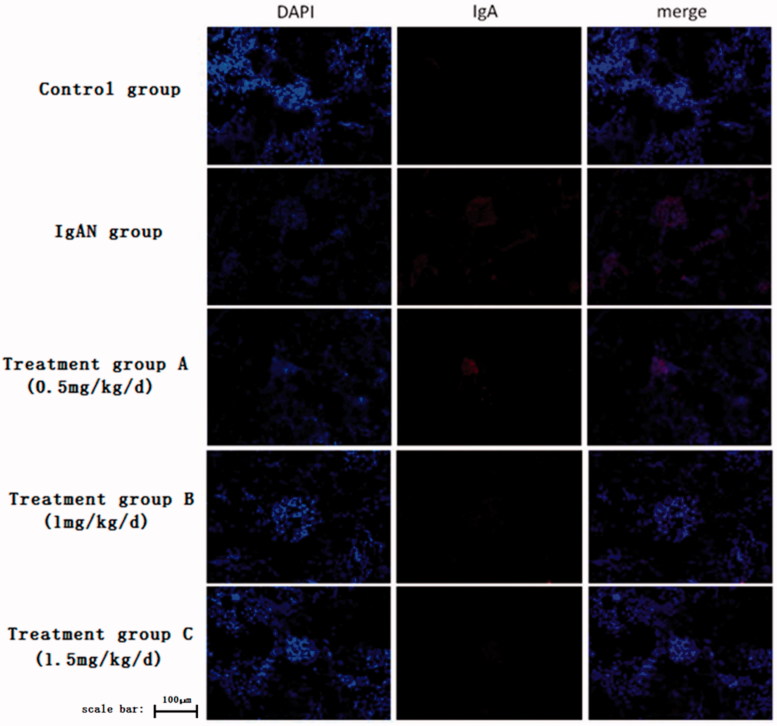
The photograph of immunofluorescence assay after different concentrations of rapamycin treatment.

The fluorescence immunofluorescence results were analyzed by Image-Pro Plus, which indicated that the IgA expression level in the normal group was very weak while it is higher in the control group. The expression of IgA decreased gradually with the dose of rapamycin increased, suggesting that rapamycin has protective effect on the IgAN model, and the protective effect depended on the dose of rapamycin under the experimental concentrations (Figure 2).

**Figure 3.**
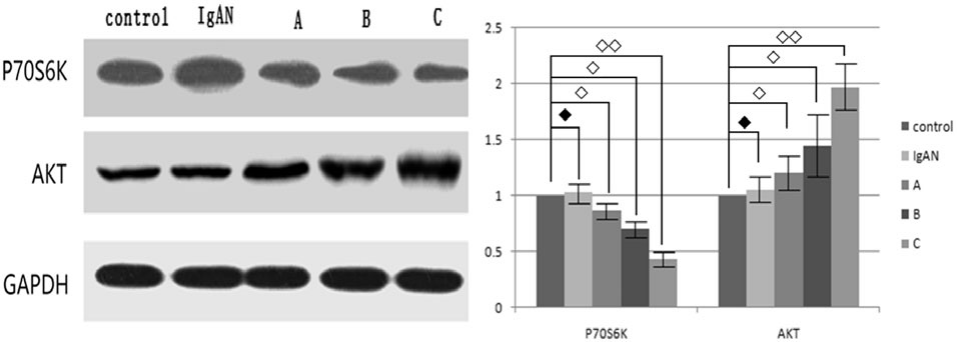
Expression of p70S6K and AKT after different concentrations of rapamycin treatment in the rats model (◆ showed there was no statistical significance, ◇ showed there was statistical significance and *p* < 0.05, ◇◇ showed there was statistical significance and *p* < 0.01).

Western blot experiment indicated that the main target of rapamycin is still the mTOR pathway. After the application of rapamycin, the expression of downstream protein p70S6K in the mTOR pathway was decreased and showed a dose relationship with rapamycin. The mTOR pathway upstream protein AKT showed up regulated and also showed a dose relationship with rapamycin (Figure 3).

## Discussion

IgA nephropathy is the most frequent form of primary glomerulonephritis that occurs in China and worldwide, and it is one of the most important causes of end-stage renal failure [10]. The etiology of IgAN remains unclear and is characterized by deposition of the IgA in the glomeruli, leading to diffuse mesangial proliferation. The clinical manifestations of IgA nephropathy varied, including repeated macroscopic hematuria or microscopic hematuria, hypertension, chronic renal insufficiency, nephrotic syndrome and end-stage renal failure [11,12].

The mTOR contains several catalytic structural domains and performs its biological functions in cells through protein-protein complexes, which are formed by the different domains of mTOR and the related proteins, and the two main types of mTOR complexes (mTOR-Raptor and mTOR-Richtor complexes) have been identified at present [13].

The interaction between the two types of mTOR complexes and their upstream and downstream signaling molecules is different. Rapamycin can inhibit the mTOR in the mTOR-Raptor complex but show no inhibitiory activity in the mTOR-Richtor complex [14,15]. The upstream of mTOR is mainly regulated by two signal pathways, namely the PI3K/Akt/mTOR and the LKB1/AMPK/mTOR signaling pathways. The mTOR is phosphorylated through the upstream signaling pathway, producing a response that regulates two different downstream signaling pathways, including the 4EBP1 and ribosomal S6 protein kinase (S6K) pathways, which control the translation of specific subunit of mRNAs, respectively [16]. The mTOR affects the function of T-cells by affecting the proliferation, growth, maturation, and protein synthesis of T-cells directly.

In this study, we successfully established a rat model of IgA nephropathy, which can be identified from the proteinuria, creatinine changes, IgA glomerular fluorescence deposition and morphological analysis of the rats in the model group. We observed the renal histological changes and expression of AKT and p70S6K in renal tissues of rats with different concentrations of rapamycin. After treatment with rapamycin, the rats showed reduced proteinuria and IgA fluorescent intensity along with increasing doses, suggesting that rapamycin can alleviate the disease severity in this IgA nephropathy model in a dose-dependent manner. What’s more, the expression of p70S6K in the downstream of the mTOR pathway was decreased and the upstream protein AKT was over-expressed, both showing with dose-dependent manner, indicating that rapamycin may play its role through the signal pathway of AKT-mTOR-p70S6K. Generally speaking, these results may suggest that rapamycin delays and fight against the occurrence and development of IgAN. However, in this experiment, all rats were sacrificed at 16th week, so the experimental data showed that the renal protective effect of rapamycin has a positive correlation with its concentration, whether the high concentration of rapamycin will produce biological toxicity to rats or the long-term effect still needs further investigation.

This study suggests that mTOR pathway is activated and participates in the development of IgA nephropathy, which means mTOR pathway might be an important target for IgAN treatment. We found that rapamycin can improve renal function and delay the progression of IgA nephropathy as a specific mTOR pathway antagonist. This finding may shed new lights for the future treatment of IgA nephropathy.
